# Two Decades of Invasive Western Corn Rootworm Population Monitoring in Croatia

**DOI:** 10.3390/insects9040160

**Published:** 2018-11-10

**Authors:** Martina Mrganić, Renata Bažok, Katarina M. Mikac, Hugo A. Benítez, Darija Lemic

**Affiliations:** 1 Department for Agricultural Zoology, Faculty of Agriculture, University of Zagreb, Svetošimunska 25, 10000 Zagreb, Croatia; rbazok@agr.hr (R.B.); dlemic@agr.hr (D.L.); 2 Centre for Sustainable Ecosystem Solutions, School of Biology, Faculty of Science, Medicine and Health, University of Wollongong, Wollongong 2522, Australia; kmikac@uow.edu.au; 3 Departmento de Recursos Ambientales, Facultad de Ciencias Agronomicas, Universidad de Tarapaca, 1000000 Arica, Chile; hugobenitezd@gmail.com

**Keywords:** western corn rootworm, population genetics, microsatellites, mitochondrial DNA, geometric morphometrics, Croatia, Europe

## Abstract

Western corn rootworm (WCR) is the worst pest of maize in the United States, and since its spread through Europe, WCR is now recognized as the most serious pest affecting maize production. After the beetle’s first detection in Serbia in 1992, neighboring countries such as Croatia have established a national monitoring program. For more than two decades WCR adult population abundance and variability was monitored. With traditional density monitoring, more recent genetic monitoring, and the newest morphometric monitoring of WCR populations, Croatia possesses a great deal of knowledge about the beetle’s invasion process over time and space. Croatia’s position in Europe is unique as no other European nation has demonstrated such a detailed and complete understanding of an invasive insect. The combined use of traditional monitoring (attractant cards), which can be effectively used to predict population abundance, and modern monitoring procedures, such as population genetics and geometric morphometrics, has been effectively used to estimate inter- and intra-population variation. The combined application of traditional and modern monitoring techniques will enable more efficient control and management of WCR across Europe. This review summarizes the research on WCR in Croatia from when it was first detected in 1992 until 2018. An outline of future research needs is provided.

## 1. Introduction

### Invasive Western corn rootworm (WCR)

Western corn rootworm (WCR) *Diabrotica virgifera virgifera* LeConte (Coleoptera: Chrysomelidae) overwinters in the egg stage in soil and emerges in spring from mid-May to early July [1,2]. The main damage to maize plants is caused by larval feeding on the roots, affecting key plant physiological processes [3]. The resulting injury leads to stalk lodging and yield losses, which further leads to economic levels of damage to maize crops.

*D. v. virgifera* was first detected in Europe in Serbia during 1992 [4], but it is suspected that the WCR began its invasion of Europe ca. 1980; however, the pest was not officially recorded until 1992 [5,6]. Once introduced, *D. v. virgifera* started to spread across Europe. Five separate introductions of the WCR from North America into Europe are known to have occurred since 1998. WCR were introduced into Northeast Italy: Veneto in 1998, Pordenone in 2002, Udine in 2003 [7], Northwest Italy and Switzerland in 2000 [7], near Paris (France) in 2002 and 2004, and in 2003 at locations in Eastern France, Switzerland, Belgium, the United Kingdom, and the Netherlands [7]. Although the invasion history of WCR in Europe is now well known, the native populations of the Western European outbreaks are still unknown [7,8,9]. Given the sequence of outbreaks, Central Southern Europe (CSE) has generally been assumed as the source of most of the Western European populations [10]. However, each outbreak could have a source population from North America, CSE Europe, or some other Western European geographic locations [10].

The invasion of Europe by the WCR occurred in three phases since the 1980s. The first phase was the accidental introduction of WCR into Europe, which occurred ca. 1980–1992 [11]. The second phase was the establishment of WCR in countries surrounding the introduction location ca. 1995–2000 (Croatia, Bosnia and Herzegovina, Hungary and Romania) [11]. From 1995 until 2001, newly invaded fields were routinely identified in this part of Europe. The final phase of the invasion (2001–2018) was the dispersal phase, where WCR spread from Serbia to occupy 22 European countries spanning tens of thousands of hectares of maize fields [11]. In subsequent years from 2002 to 2011, WCR population densities have been relatively stable in all areas of maize production where their reproduction (an indicator of an established population) has been stable [8]. Evidently, established WCR have been spreading since their original introduction (ca. 1995), and as such, the more recent invasion phases of establishment and spread co-exist in Southern Europe [2].

During all stages, different monitoring techniques have been conducted in Croatia to detect, estimate and predict WCR population abundance and annual variations. In this review, we present traditional population metric surveys that were conducted in the first years of the WCR invasion in Croatia, and modern monitoring techniques, such as population genetics and geometric morphometrics, which were subsequently used to provide information on the variation within and among WCR populations. The monitoring techniques and procedures used in Croatia since the 1990s were implemented to inform management practices and contribute data to the effective integrated pest management (IPM) of WCR and other invasive pests in agricultural production.

## 2. Monitoring Trap Methods

Formal WCR monitoring in central European countries started in 1996. This initiative was undertaken by the International Working Group on *Ostrinia* and other maize pests (IWGO) as part of the International Organization for Biological and Integrated Control (IOBC) to organize and facilitate international collaboration. The first international meeting was held in Graz, Austria (20–21 March 1995) where the decision was made to start a monitoring program in countries at risk of WCR invasion and determine suitable control methods [11]. Soon after this meeting, the first formal survey and detection of WCR adults in Croatia [3,12,13,14,15,16,17,18,19,20,21,22] and in Hungary [23] were completed in 1995. Since then, IWGO has organized regular international conferences to report on the status of WCR and the associated research completed in Central and Eastern Europe. Due to these meetings and the associated reporting framework, WCR has become the only insect pest in the world whose monitoring and spread have proceeded in different areas and have been documented using the same methodologies. In the initial phase of WCR monitoring, cucurbitacin traps were implemented; however, pheromone traps developed by Hungarian researchers were found to be suitable for the early detection of a pest population. The usefulness of the pheromone traps was quickly realized and as early as 1996, all the monitoring actions in all the invaded countries used pheromone traps. However, yellow sticky traps have been used, especially when WCR population levels exceeded a threshold [15].

The results of research activities on WCR in Europe and the USA were presented during IWGO conferences [11]. Adult WCR monitoring by European countries allowed for the rapid detection and consequent understanding of WCR invasion processes since their first detection in Serbia [4,5,8]. The success of WCR monitoring and research in Europe resulted from the establishment of permanent monitoring sites in network partner countries, i.e., Serbia [24], Hungary [8], and Italy [25]. Permanent monitoring sites have allowed for the measurement of population fluctuations over the years. Between 1996 and 2006, the monitoring of WCR was regularly conducted in invaded and non-invaded areas in Croatia. The aims of the monitoring activities were to establish the rate of spread [14,18] and route [17] of WCR across Croatia, to evaluate the attractiveness of pheromone traps vs. yellow sticky traps [22,26,27,28,29], to document the flight dynamics of WCR adults [30], and WCR population changes over time [31]. This research was undertaken with the aim of assisting with their ongoing integrated management.

## 3. Spatial and Density Monitoring

Soon after WCR detection in Serbia, Maceljski and Igrc-Barčić [32] studied the biology and ecology of WCR and the potential for its establishment in Croatia. Preliminary studies on these areas showed that WCR would likely survive and develop wherever maize is grown in Europe [33]. WCR monitoring started in Croatia and in surrounding countries specifically for its detection and dispersion compliance [17,34,35,36,37]. During the monitoring period, four types of attractant traps were used: cucurbitacin traps, pheromone traps, Pherocon^®^ AM (PhAM) non-baited yellow sticky traps (Treece, Salinas, CA, USA), and Multigard^®^ (Sentry, Billings, MT, USA) non-baited yellow sticky traps. The first cucurbitacin trap designed for capturing *Diabrotica* spp. was constructed from amber plastic vials measuring 3 cm in diameter and 9 cm in length [38]. Pheromone traps are baited with synthetic sex pheromones and only catch males; they are highly sensitive tools for detection of occurrence and general monitoring. The sticky sheet is transparent and has a catch capacity of 3–400 beetles [39]. PhAM and Multigard^®^ are yellow sticky surface traps used to monitor WCR. The color of the trap is visually attractive to the pests [40]. In the first years of monitoring, Multigard^®^ yellow sticky traps were used, but in 2000, they were replaced with the PhAM trap. The switch was made to enable comparison with U.S. monitoring data [31].

**Figure 1:**
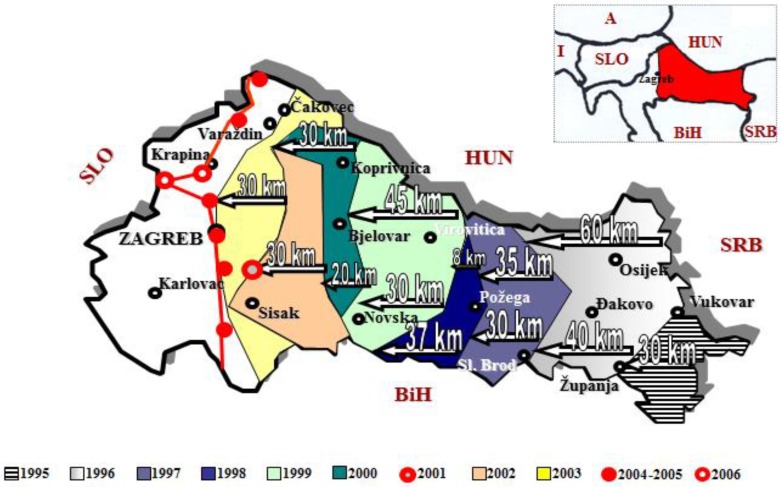
Distribution of western corn rootworm (WCR) in Croatia, established using spatial and density monitoring techniques.

During 1995, the first year of monitoring, 150 baits from USA with low attractant cucurbitacins, were placed in maize fields in Croatia. Cucurbitacin traps were really acting as a feeding arrestant, rather than attractant, because they are small tubes with dry plant material inside, and the plant material come from Cucurbita spp., rich in cucurbitacin. This compound is a feeding stimulant for WCR, so it keeps the beetles coming to the trap, but does not attract them [38]. As a result of this intensive monitoring process, one WCR specimen was caught in Bošnjaci near the border with Serbia, this was the first detection of WCR in Croatia [12]. Since 1996, the Department for Agricultural Zoology at the Faculty of Agriculture University of Zagreb, supported by the Croatian Ministry of Agriculture and Forestry, formally organized and undertook the WCR monitoring activities in Croatia [37]. After the first WCR detection in Europe, a pheromone lure was produced by European scientists and pheromone traps for monitoring purposes were designed. This trap was used in Croatia during the period between 1996 and 2006 [33]. Monitoring was conducted in seven Croatian counties during 1996, and in eight counties during 1997, 1998, and 1999. According to Igrc-Barčić and Dobrinčić [17], in 1996, the beetle spread 80 km to the west of the initially invaded sites and further infested 6000 km^2^ of the maize production area; in 1997, the beetle infested approximately 9000 km^2^ of the maize production area. In 1998, movement of the WCR to the west was less than recorded in the previous three years. The only movement of the beetle was recorded along the river Sava. From 2000 to 2002, monitoring was conducted in 11 counties, this increased to 13 counties from 2003 to 2005, and in 2006, monitoring occurred in 11 counties. Each year, traps were set in maize fields (between 31 and 148 fields/year) situated in different areas of Croatia where the beetle could be found. Together with Pherocon^®^ AM (PhAM) pheromone traps, non-baited yellow sticky traps (Treece, Salinas, CA, USA) or Multigard^®^ (Scentry, Billings, MT, USA) non-baited yellow sticky traps were installed [31]. Economic damage levels in maize, resulting in an 85% reduction in yield, were observed in the Baranja region in Croatia during 2002, which is 200 km from Surčin, Serbia, the site where the WCR was first introduced into Europe [3]. During the 11 years of WCR monitoring in Croatia, it was possible to accurately predict the direction and intensity of the spread of WCR for the following year. From the data gathered, WCR spread at a rate of between 20 and 60 km/year in a westerly direction through Croatia, which acted as a corridor for the beetle’s dispersal into the rest of Europe (Figure 1) [37].

Of all the traps evaluated, pheromone traps were most sensitive for early detection purposes. They were used not only to predict the line of spread, but also to describe the flight and population dynamics in a continuously sown maize field (a prelude to research on crop rotation as a mechanical control) [31]. Pheromone traps were also used to measure how far WCR adults would travel into neighboring fields for oviposition. WCR adults were monitored in continuous maize fields in 2003 and 2005 using Pherocon^®^ AM non-baited yellow sticky traps [41]. Adult WCR population densities in 30 cornfields were determined weekly over a 74-day period each year (from the 24th to 35th week of the year) during 2006–2009 [42]. Adult population density was established in the 29th week of the year. At that time, the maize phenology stages varied from R65 to R67, according to the BBCH scale [43].

Pheromone trapping enabled efficient WCR occurrence and population abundance monitoring and the prediction of potential damage to maize crops during the following year [22,44]. According to Bažok et al. [22], a potential substitute for the Pherocon^®^AM trap is the “whole plant count” method used in the first half of August. The Pherocon^®^AM trap/week capture corresponds well with the whole plant count method. Both methods can be used to estimate adult WCR population density. WCR larvae are present in the soil during the maize phenology stage from R18 to R34 according to the phenological growth stages and the BBCH maize identification keys [43]. Larval infestation was best predicted by maximal weekly capture; however, root damage was better predicted by the capture of adults in the 31st week of the previous year [45]. To predict plant lodging, three parameters were found to be equivalent in their predictive ability: maximal weekly capture; average daily capture; and the capture of adults in the 29th week of maize production [42]. Plant lodging was estimated in the 38th week of the year. At that time, the maize phenology stages varied from R83 to R97 according to the BBCH scale [43]. Larval emergence was predicted by the observed number of adults and eggs in the year preceding repeated maize sowing [2,45,46]. The highest density of Croatian WCR populations was recorded in 2003, when the average number of adults was *n* = 1275 and *n* = 177 on pheromone traps and yellow sticky traps, respectively. The relationship between the average number of adults captured per trap and climatic conditions (mean weekly temperature and rainfall) from weeks 25 to 35 of the year was estimated during 2007–2009. The average number of WCR per field was highest in years with higher amounts of rainfall and lower summer temperatures. Regression tree analyses showed that total rainfall was the best predictor of WCR population abundance [2]. The identification of the most important habitat parameters for WCR enabled predictions of infestation and potential levels of annual damage with the main purpose of informing farmers about the most efficient control strategies [45,46].

Traditional population surveys are important in WCR IPM, and can be effectively used to predict WCR population abundance [47]. Pheromone traps are more suitable for the monitoring and prediction of population increase, but for scouting purposes, yellow sticky traps are more a better option. Determining the factors that positively or negatively affect WCR population abundance in some regions is the starting point for the development of IPM strategies on a national and international scale.

## 4. Genetic Monitoring

In Croatia, the historical and contemporary population genetic structure of WCR was investigated from 1996 until 2009 [48,49,50]. This was the first study to use the temporal and spatial genetic structure to estimate the diversity, gene flow, invasion dynamics of WCR in Croatia and the influence of control practices on these population genetics parameters [51,52]. From the more than 1500 adult WCR investigated from 1996 to 2009, six microsatellite markers revealed that one large WCR population existed in Croatia in 1996 and in 2009. While the population changed over time, microsatellite markers revealed the persistence of a single large population.

Deciphering the temporal and spatial genetic structure of WCR has had important implications for the IPM of this invasive pest. By investigating WCR across Croatia over a 13-year period, it was possible to determine that in the absence of control (during 1996–2009), genetic diversity increased and minimal genetic structure remained, even to this day. Through crop rotation control practices, the WCR population should respond with a decrease in the genetic diversity of the populations/individuals under investigation as well as a noted increase in genetic structure. The genetic structure should then act to fragment or sub-structure and isolate populations geographically thus restricting gene flow. Ciosi et al. [9] found a pattern of isolation by distance, suggesting that the spreading population in Eastern Europe was split into genetically differentiated populations. Despite this, lower genetic diversity has not hampered the invasion and spread of WCR in Croatia, with 85,000 ha [15] of maize crops infested in 1996 compared with the 295,000 ha infested in 2007 [41]. A single panmictic population characterizes the overall population genetic structure of WCR in Croatia [50].

In addition to nuclear microsatellites, mitochondrial DNA markers have been used to monitor WCR population genetics on a microgeographic scale in Croatia [53]. This was the first study to formally conduct genetic monitoring of WCR through the use of multiple markers. Specifically, microsatellite markers were used to investigate the genetic variability and structure of the WCR collected in 1996, 2009, and 2011 from numerous locations across Croatia, Serbia, and the U.S. The study also reported bottleneck events and the location of the geographic source of WCR in Croatia (i.e., Serbia).

Ivkosic et al. [53] demonstrated that the seven U.S. WCR populations investigated maintained the greatest allelic diversity when compared to Croatian and Serbian WCR. In Europe, the largest number of alleles was found in locations near international airports (Rugvica, Croatia and Surčin, Serbia). The highest number of mtNDA haplotypes was observed in Croatia in 1996, soon after WCR was first recorded there. From 2009 to 2011, haplotype diversity declined, and Croatia and Serbia had one fixed haplotype. Furthermore, continuous maize cropping locations in the U.S. had one haplotype, whereas three haplotypes were found in soybean-maize crop-rotated locations. Minimal temporal genetic variability was found among the populations in Europe and the U.S.; a result previously demonstrated for the species only in the U.S. [54]. Bayesian cluster analysis revealed two genetic clusters that joined the WCR from Croatia and Serbia, but separated them from U.S. populations. These clusters showed that numerous U.S. individuals had both European and U.S. ancestry, which suggests the existence of bidirectional gene flow [55]. Bottlenecks were identified within all Croatian populations sampled in 1996 and 2011 and only two populations in 2009. Bottlenecks were not identified at all in Serbia from 1996 to 2011, or in the U.S. in 2011. As suspected, Serbia was revealed as the geographic source of WCR in Croatia. The temporal genetic monitoring conducted from 1996 until 2011 allowed a deeper understanding of the WCR genetics in Croatia, Serbia, and its original geographic region in the U.S.

More recently, the population genetics of WCR in Southern Europe during all invasive phases (introduction, establishment, and spread) were investigated [55]. Results from the study showed that during the first phase (introduction), the number of observed alleles was low (19–27; 45%) in Southern Europe compared to suspected source populations from the U.S. (Iowa or Illinois). Within a relatively short time period, the number of alleles present in Southern Europe approximately doubled. Of all known WCR alleles [54,56], 84% were found in locations in Southern Europe, 14 years after WCR was first introduced. During the second and third invasive phases (establishment and spread, respectively), the number of alleles in the population in Croatia had doubled compared with the other countries investigated in the study. However, this may have been due to the intensive monitoring program in Croatia during the study period [50]. The results confirmed the original finding that allelic richness during the introduction phase was low but consistent throughout all Southern European populations [55]. However, during the establishment and spread phases of the invasion process, allelic richness was higher for all Southern European populations. Croatian populations in the same period had significantly higher allelic diversities than any other European population investigated. These analyses revealed previously undiscovered alleles during the invasive phases of WCR in Europe. Specifically, two unique alleles were found in the introduction phase, whereas nine previously unrecorded alleles were found during the establishment and spread phases. The large number of unique alleles found in this study could reflect multiple and ongoing invasions in Southern European countries from different locations within Europe and the U.S. These results confirm that Serbia was the primary source of WCR to its neighboring countries (Croatia, Hungary, and some parts of Italy). The only exception to this was the WCR population in Venezia, Italy, which was formed after a second introduction from the U.S. [55].

A detailed population genetics investigation of the WCR invasion phases (introduction, establishment, and spread) conducted by Lemic et al. [55] revealed that the three phases often overlap and that these phases of invasion are still in progress in Europe. Extensive population genetic investigations of WCR in South Europe have revealed that low genetic variation exists among the populations in Italy, Austria, Hungary, Slovenia, Croatia, and Serbia, and showed minimal genetic differences between populations and among regions [55,57].

For over a decade, population genetic monitoring has been used to inform the effective control and ongoing integrated management of invasive WCR in Croatia [58] and has proven useful in understanding WCR invasion in Croatia and other invaded countries. The results obtained from these studies are crucial to further understand WCR population dynamics during the major phases of its European invasion [57]. An investigation into the WCR’s population genetic structure, gene flow, and dispersal patterns has helped to understand the impact this invasive species has had on global agriculture production and food resources.

## 5. Geometric Morphometric Monitoring

The expense and need for specialist skills associated with population genetics were the main reasons to search for additional non-genetic based techniques to monitor WCR. Geometric morphometrics (GM) were tested and deemed an existing novel use method to easily, cheaply, and quickly yield robust data. After almost two decades of traditional (distribution and abundance) and genetic monitoring of WCR populations in Croatia, geometric morphometric monitoring was used with the aim of assessing whether WCR wing shape and size were influenced by specific habitat parameters that could enable the discovery of a population biomarker [55].

In the application of the technique to understand invasion patterns in WCR, Mikac et al. [59] were the pioneer researchers to include GM in IPM research for WCR. These authors demonstrated discernable patterns in wing size and shape between resistant (crop rotation) and susceptible populations in the USA. Their research provided the foundation for and set the research agenda of GM use in WCR IPM research that has since followed [2,57,58,60,61,62,63].

Following Mikac et al. [59], Lemic et al. [57] and Benítez et al. [61] showed that GM could be used as a tool to examine wing shape differences influenced by environment. These authors tested their hypotheses in WCR populations principally from Croatia, where varying soil types are known to directly influence larval and adult WCR development [41]. Both Lemic et al. [57] and Benítez et al. [61] demonstrated that WCR wing shape changed according to major soil type classifications in Croatia. These results were novel for WCR and a need to further test these findings drove the research questions of subsequent similarly themed work.

For example, Lemic et al. [57] compared the hindwing shape and size between sexes of WCR from populations sampled in the U.S. and Europe. The populations investigated showed high levels of sex wing shape dimorphism [57]. Both in the U.S. and Europe, female WCR had more elongated wings. Since elongated wings are considered to be involved in migratory movement, this investigation provided morphological evidence that most migration in WCR (as well as invasive migrations) could be attributed to the females of this species. Female WCRs are also known to undertake migratory flights over relatively long distances. This was also discussed by Mikac et al. [59], who suggested that elongated wings were probably more aerodynamic and may be a useful invasive dispersal strategy for mated females. When investigating sexual dimorphism within a species, it is also important to examine whether allometry contributes to sexual dimorphism [62,64]. Allometry is the relationship between size and shape and is normally categorized as a percentage where shape is explained by size. Insect studies of allometry are normally related to the nutritional aspect to which development is directly related [65]. In addition to the described results, the presence of asymmetries in the WCR wings is a novel finding for coleopterans and is an important contribution to the ever-growing pool of data on the evolution of insect wings [61].

Morphological integration and modularity are another set of analyses that can be performed using GM tools to infer the developmental structure of morphology [66] and to answer questions about the invasiveness of WCR. Benitez et al. [62] analyzed the relationship among landmarks in the hindwings of WCR to explain why their wing structure is composed of different modules. Surprisingly, the results showed an integrated behavior of the hindwings of WCR. These findings paved the way for future flight performance and biogeographical studies on how wing shape and size change across the native and newly invaded range of WCR in the U.S. and Europe [62].

Two years later, Mikac et al. [63] confirmed that GM tools were again useful to identify invasion processes (i.e., multiple WCR introductions into Europe) for the WCR and could be used as a special monitoring tool for this pest species. This research studied the hindwing size and shape variations within and among WCR populations over a larger geographic region in Southern Europe, spanning an area of 160,000 km^2^. The data generated represent the greatest morphological investigation of an invasive species with global importance. The results allowed the WCR populations from Italy and those in Central and Southeastern Europe to be clearly separated [6,64], a result mirrored in Lemic et al. [55] who demonstrated the same result using population genetic markers. Additionally, the wing shape differences found using GM procedures followed an east to west direction of spread as described by Igrc-Barčić et al. [37]. Based on genetic [55] and now GM data [63], it was possible to conclude that the Italian WCR population had no link to the aforementioned populations and originated from a different and more recent introduction from the U.S. Notably, although the conclusion on genetic monitoring required two decades of WCR analysis [55], through the use of GM monitoring, valuable information on the invasion process was obtained from the analysis of WCR in a single time period (i.e., here in 2012).

Most recently, Mikac et al. [60] extended the use of hindwing size and shape differences to examine changes in WCR related to the development of resistance, specifically investigating possible differences among rotation resistant, *Bacillus thuringiensis* (*Bt*)-resistant, and non-resistant (or susceptible) populations in the U.S. In general, the hindwings of non-resistant beetles were significantly more elongated in shape and narrower in width (chord length) in comparison to beetles that were resistant to *Bt*-maize or crop rotation. Such differences may impact the dispersal or long-distance movement of resistant and susceptible WCR, as wing morphology is a critical element of an insect’s dispersal capacity. Understanding which morphotype of the beetle is the superior flier and disperser has implications for the management of WCR via integrated resistance strategies. Overall implications from the GM work conducted to date suggest that GM can be used to monitor population changes related to the invasion process and could be used as a cheaper and more accessible population biomarker compared to expensive and specialized-use genetic markers when investigating biological invasions in species that have similar characteristics to WCR.

## 6. Future Work

In an effort to broaden our understanding of WCR invasion biology and the response to integrated management practices, genetic and phenotypic methods must be investigated. Currently, the use of single nucleotide polymorphisms (SNPs, pronounced ‘snips’) in non-model organisms has become an affordable and readily accessible means of generating important data on species that otherwise would have been impossible due to cost and expertise availability. For use in population genetics, SNPs have surpassed microsatellites as the marker of choice, and using them to understand the population genetics of WCR on a deeper level must be explored. The use of SNPs as population genetic marker in WCR has been attempted, though only a limited number of individuals (*n* = 12) were genotyped and the results were similar to those from microsatellites [67]. Given the latest technology in next generation sequencing and the now routine use of genotyping by sequencing SNPs, the potential for robust and plentiful population genomic data to understand WCR movement patterns on small and large geographic scales warrants investigation. Finally, future work on phenotypic aspects of WCR are needed to compliment any population genomic data that is generated. In particular, a greater understanding of WCR intraspecific flight morphology is needed to better understand the fundamentals of WCR dispersal. Our findings on the changing WCR hindwing shape and size, according to resistance, has provided researchers and managers alike with important morphological information on resistant morphotypes on which monitoring can focus. A deeper understanding of WCR wing shape and flight morphology, aspect ratio, and flight efficiencies will assist with the management of the species. Such information is crucial for the implementation of biosecurity measures and integrated pest management strategies for the WCR globally.

### List of Projects Related to WCR in Croatia

- 2017–2021: Monitoring of insect pest resistance: novel approach for detection, and effective resistance management strategies (MONPERES), Croatian science foundation (coordinator: R. Bažok)
- 2009: The landscape genetics of the invasive western corn rootworm in Croatia (Ministry of science, education and sport—Unity through knowledge fund—UKF)
- 2007–2013: The spatial distribution of economically important pests with the use of GIS (Ministry of science, education and sport, Croatia)
- 2005–2007: Developing IPM in maize through WCR risk management—FAO
- 2005–2006: Development of IPM for WCR in collaboration with Secondary agricultural schools-FAO
- 2003–2007: Integrated pest management for western corn rootworm in Central and Eastern Europe (FAO, GTF)
- 2002–2006: Biological control the base of ecologically acceptable plant protection (Ministry of science and technology, Croatia)
- 2002–2004: The possibility of the control of the Western corn rootworm with minimal input (Ministry of agriculture and forestry Croatia)
- 1998–2006: Monitoring of the western corn rootworm (Ministry of agriculture and forestry Croatia)
- 1998–2001: *Diabrotica virgifera virgifera* (Ministry of science and technology Croatia—young researcher project)
- 1997–2000: Management of Western corn rootworm in central Europe FAO/TCP

## 7. Conclusions

The thorough knowledge of the WCR invasion in Croatia is unique in Europe, as no other European nation has demonstrated such a detailed and complete understanding of an invasive insect till now. This review summarized the research on WCR in Croatia from 1992, when it was first detected, until 2018. It outlines the important work undertaken on multiple aspects of WCR biology, ecology, population genetics and morphometrics to inform integrated pest management strategies used for its effective control. Early stages of the research focused on the detection and monitoring of the beetle using traditional methods (yellow sticky traps etc.) and then progressed to genetic monitoring (microsatellites and mitochondrial DNA markers) of Croatian and wider European populations of WCR. The most recent research on WCR in Croatia has focused on the use of geometric morphometrics as a monitoring tool and population biomarker. Given the very detailed understanding of the biology, ecology and genetics of WCR that Croatia has, it is very well placed to effectively detect, monitor and control WCR within its borders.
